# Data resource profile: JMDC claims databases sourced from Medical Institutions

**DOI:** 10.1002/jgf2.367

**Published:** 2020-08-30

**Authors:** Katsuhiko Nagai, Takashi Tanaka, Norihisa Kodaira, Shinya Kimura, Yoshimitsu Takahashi, Takeo Nakayama

**Affiliations:** ^1^ JMDC Inc. Tokyo Japan; ^2^ Department of Health Informatics Kyoto University School of Public Health Kyoto Japan

**Keywords:** database, DPC, health insurance, Japan, medical institutions

## Abstract

JMDC, Inc. (JMDC) has created a database, using data collected from medical institutions in Japan, consisting of claims (for hospitalization and outpatient treatment), diagnosis procedure combination (DPC) assessment forms, and clinical laboratory test values. The oldest data in this database that can be accessed relate to treatment in April 2014. Currently (the end of October 2019), the number of medical institutions is 218, consisting of 131 DPC‐eligible hospitals and 87 DPC‐ineligible hospitals. Using this database, it is possible to carry out an analysis that makes up for certain limitations of JMDC's another database of data from health insurance societies (eg, the disease status and test results cannot be ascertained, and there is insufficient access to data for elderly people). In addition, it is noteworthy that this database includes not only data from DPC‐eligible hospitals but also data from some DPC‐ineligible hospitals.

## Graphical abstract

JMDC, Inc. (JMDC) has created a database, using data collected from medical institutions in Japan, consisting of claims, DPC assessment forms, and clinical laboratory test values. Using this database, it is possible to carry out an analysis that makes up for certain limitations of JMDC's another database of data from health insurance societies.

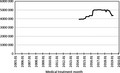

## 1 DATA RESOURCE BASICS

Japan has health insurance provided by the social insurance system^1^ (referred to below simply as "health insurance"), and when a citizen is examined and/or treated at a medical institution, he/she presents to that institution a health insurance certificate containing information identifying him/herself and his/her insurance provider, and he/she is then responsible for only a fixed proportion of the medical expenses. The medical institution invoices the insurance provider for the remainder of the sum, via a claims processing and payment organization, on the basis of information in the health insurance certificate. Health insurance is provided by multiple bodies, on the basis of occupation, geography, and age (eg, elderly and geriatric), as follows:^2^

- People aged 75 or older can join the medical care system for the late elderly, administered by local governments.
- People less than 75 years old, primarily those who are employees of small, medium‐sized, or long‐established businesses, and/or are in temporary employment, and their dependents, are covered by health insurance administered by the Japan Health Insurance Association, except for employment‐related injury.
- People less than 75 years old, primarily those who are employees of large businesses, and their dependents, are covered by health insurance administered by health insurance societies, except for employment‐related injury.
- Public‐sector employees and private school teachers/staffs who are less than 75 years old are covered by health insurance administered by the National Public Service Mutual Aid Association and the Promotion and Mutual Aid Corporation for Private Schools of Japan.
- Seamen less than 75 years old, and their dependents, are covered by seamen's insurance administered by the Japan Health Insurance Association, except for employment‐related injury.
- People, primarily nonmanual workers, who are less than 75 years old are covered by national health insurance administered by local governments.

JMDC, Inc. (JMDC) has created a database, detailed below, using data collected from medical institutions.

**FIGURE 1:**
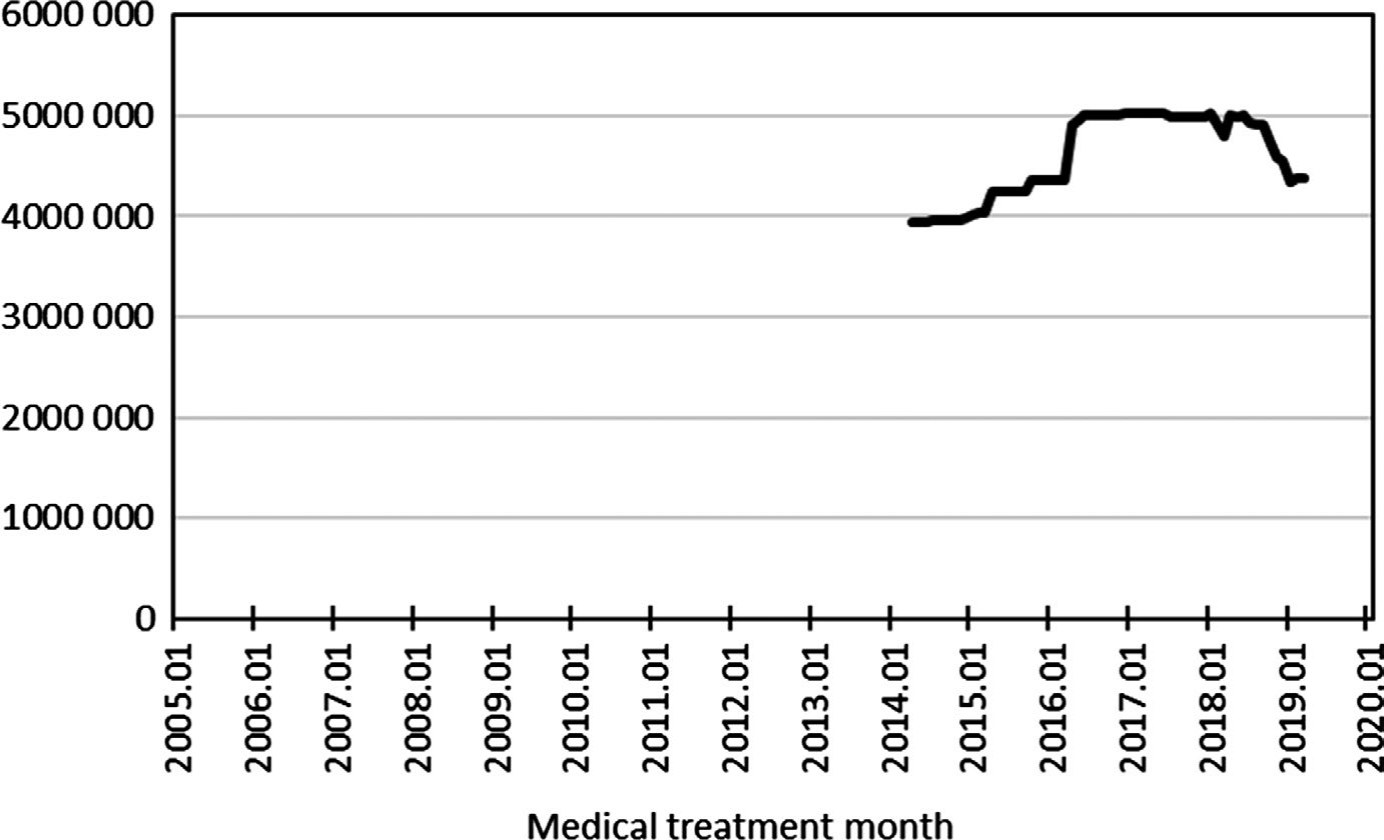
Numbers of patients whose data can be accessed each month

JMDC has established this database using data collected from multiple Japanese medical institutions:

1. The collected data consist of claims (for hospitalization and outpatient treatment), diagnosis procedure combination (DPC) assessment forms (Form 1 and EF file and H file), and clinical laboratory test values. The database is constructed using merged data, by combining these collected data. The claims include information about medical expenses for which invoices are sent to insurance providers by medical institutions. The DPC assessment forms are documents that the Japanese Ministry of Health, Labor, and Welfare (MHLW) requests medical institutions to provide in order to assess the level of effects, in connection with the DPC, which consists of comprehensive evaluation of diagnostic categories and is a medical expense calculation method for defining daily hospitalization expenses for each diagnostic category, and these forms consist of the following: (a) Form 1, which contains detailed information about hospitalization, in a 'a medical records' format; (b) EF file, which contains itemized information about medical expenses, similarly to the claims; and (c) H file, which contains information about the nursing care requirement level. The clinical laboratory test values were the results of tests based on version 10 of the Japanese Laboratory Code (JLAC10), which was defined by the Japanese Society of Laboratory Medicine.
2. JMDC provides services of aggregating and analyzing clinical quality indicators to medical institutions distributed throughout Japan. Some of these institutions agree to provide anonymously processed data which do not include area information to third parties. Data are collected from these medical institutions.
3. Data are collected monthly, and the collected data are added to the database approximately 1 month after the treatment date. Data are currently still being added to the database.
4. In order to protect personal information, data from medical institutions are collected as information that has been anonymized in accordance with Clause 2:9 of the Law for the Protection of Personal Information, on the basis of a personal ID for each medical institution with which individuals cannot be identified.
5. The oldest data in the database that can be accessed relate to treatment in April 2014. All items of claims, DPC assessment forms, and clinical laboratory test values are included from April 2014.
6. Currently (the end of October 2019), the number of patients included in the database, including those removed at some point, is approximately 9.4 million.
7. The numbers of patients whose data can be accessed are shown in Figure 1 for each month. The number of patients tends to increase with increasing number of medical institutions collecting data. Currently (the end of October 2019), the number of medical institutions is 218, consisting of 131 DPC‐eligible hospitals and 87 DPC‐ineligible hospitals.

Research carried out using this database includes the following:

1. Factor analysis of outcome^3^, ^4^
2. Patient characteristics^5^, ^6^
3. Validations of indices^7^

## 2 DATA COLLECTED

Any information that can identify individuals is anonymized by the medical institution, in such a way that, when the same individual is examined or treated at the same medical institution, he/she can be distinguished on the basis of the anonymized patient ID.

Nonstandardized data are standardized using a dictionary.4^8^ This is done because the terms used differ among medical institutions. For example, "type‐2 diabetes" may be entered as "diabetes II," "non‐insulin dependent diabetes," "NIDDM," or "adult‐onset diabetes." In order to overcome this problem, the JMDC uses a computer‐based, retrospective standardization method. Terms are entered from the claims without modification, after which they are standardized using the dictionary developed by JMDC. Using this method enables resolution of problems such as the following, which occur with entry of terms standardized at the entry stage:

1. It is essential to share between personnel the rules and knowledge relating to entry used in the manual procedure, and the training of personnel will, therefore, incur financial costs.
2. If terms are not reassessed once they have been entered, the latest version, based on master data updates, will not be reflected, and errors will be introduced into the time‐series analysis.

Claim data and data of DPC assessment forms entered in the database are accorded master data for classification in order to increase the effectiveness and precision of analysis. These master data for classification include the following:

1. Disease class according to the 10th revised version of the International Statistical Classification of Diseases and Related Health Problems (ICD‐10), as defined by the World Health Organization (WHO).
2. Anatomical therapeutic chemical (ATC) pharmacological activity class, as defined independently by the WHO and the European Pharmaceutical Market Research Association (EphMRA). Both of these are included.
3. Drug components
4. Drug dosage form
5. Medical institution affiliation

The clinical laboratory test values are standardized based on version 10 of the Japanese Laboratory Code (JLAC10), which was defined by the Japanese Society of Laboratory Medicine.

JMDC checks if the data received are duplicated, and if there are any unnatural fluctuations in the amount of data received each month. Input values that do not match the formats and input values outside the normal distribution ranges set by JMDC (eg, future dates) are also verified independently by the JMDC. When these values are discovered, errors are given, and they are converted into regular expressions or regular codes. Although missing values are left as they are, missing values are relatively small because the claim data submitted to the claims processing and payment organization are error‐checked in the external examination processes, and the items of the DPC assessment forms require inputs when the specified input conditions are met.

The database consists of tables for the following: (a) patients; months when data are missing; claims; claims: injuries/diseases; claims: drugs; claims: medical care activities; claims: treatment materials; Form 1; Form 1: injury/disease; EF file; EF file: drugs; EF file: medicines brought at admission; EF file: medical care activities; EF file: treatment materials; H file; and clinical laboratory test values; and (b) master data for injuries/diseases, drugs, medical care activities, etc., including standardized medical terms. Explanations of each table and examples of fields are shown in Table 1.

**TABLE 1:** Database of data from medical institutions

Table	Explanation	Field example
Patients	Patient information	Month and year of birth, gender code, first month observable, last month observable, flag for missing data
Month with missing data	Information about patient's month with missing data	Month and year with missing data
Claims	Claim information summary (data provided to claims processing and payment organization)	Month and year of medical care, code for type of claim (hospitalized, outpatients, DPC, etc), actual number of days of medical care, hospital admission date, discharge date, number of points (medical expenses), diagnosis procedure combination (DPC) code
Claims: injury/disease	Information about injury/disease of hospitalized patients and outpatients (data provided to claims processing and payment organization)	First date of medical care of injury/disease, ICD‐10 classification code, injury/disease code, flag for principal injury/disease, flag for injury/disease which used the most resources, flag for suspicious injury/disease, outcome category code
Claims: drugs	Drug information relating to hospitalized patients and outpatients (data provided to claims processing and payment organization)	Prescription date, drug preparation date, ATC classification code, WHO‐ATC classification code, MHLW drug code, drug code for electronic claim processing, daily dose per prescription, dose unit, number of days of administration per prescription, dosage, number of points (medical expenses), flag for generics, flag for drugs to take as necessary, dosage form classification code
Claims: medical care activities	Information about medical care activities of hospitalized patients and outpatients (data provided to claims processing and payment organization)	Medical care activities date, medical care activities code, number of times performed, number of points (medical expenses)
Claims: treatment materials	Information about materials for hospitalized patients and outpatients (data provided to claims processing and payment organization)	Treatment date, material code, number of times performed, number of points (medical expenses)
Form 1	Discharge summary of simplified version (data provided to MHLW)	Hospital admission date, discharge date, previous discharge date, reason for readmission, department classification code, month and year of birth, body mass index (BMI), smoking index, pregnant/not pregnant, independence degree of elderly dementia patients, nursing care requirement level, hospitalization route, brought by ambulance or not, discharge disposition, outcome at discharge, death within 24 h or not, surgery information (surgery name, date, etc.), cancer initial onset or recurrence, TNM cancer classification, cancer stage, cancer chemotherapy administered or not, activities of daily life at admission and discharge (eating, transfer movements, grooming and makeup, toilet activities, bathing, horizontal walking, climbing stairs, changing clothes, bowel control, urination control), functional independence measurement (FIM) scores at ward admission and ward discharge (eating, grooming and makeup, bed baths, upper‐body clothing change, lower‐body clothing change, toilet use, bowel control, urination control, bed/chair use, transfer to toilet, bathing/showering, walking/using wheelchair, climbing stars, communication comprehension, communication expression, social interaction, problem‐solving, cognition), Japan Coma Scale (JCS) score at admission and discharge, pre‐onset Rankin scale in stroke patients, discharge time modified Rankin scale score in stroke patients, onset time in stroke patients, Hugh‐Jones classification of respiratory disease patients, whether or not brain tumor patients treated with temozolomide, severity in pneumonia patients, New York Heart Association (NYHA) functional classification of heart failure patients, Canadian Cardiovascular Society (CCS) classification of angina and chronic ischemic heart disease patients, Killip classification of acute cerebral infarction patients, Child‐Pugh classification of hepatic cirrhosis patients, severity in acute pancreatitis patients, information about treatment of rheumatoid arthritis patients, delivery information about obstetrics patients, Burn Index in burn patients, Global Assessment of Functioning (GAF) score in psychiatric patients, Sequential Organ Failure Assessment (SOFA) score in intensive care patients
Form 1: injury/disease	Hospitalized patient injury/disease information (data provided to MHLW)	ICD‐10 classification code, injury/disease code, flag for principal injury/disease, flag for injury/disease which used the most resources, flag for suspicious injury/disease
EF file: drugs	Drug information about hospitalized patients and outpatients (data provided to MHLW)	Prescription date, drug preparation date, ATC classification code, WHO‐ATC classification code, MHLW drug code, drug code for electronic claim processing, daily dose per prescription, dose unit, number of days of administration per prescription, dosage, number of points (medical expenses), flag for generics, flag for drugs to take as necessary, flag for prescription at pharmacy, flag for generic prescription, Dosage form classification code
EF file: medicines brought at admission	Information about medicines brought at admission (data provided to MHLW)	Prescription date, drug preparation date, ATC classification code, WHO‐ATC classification code, MHLW drug code, drug code for electronic claim processing, daily dose per prescription, dose unit, number of days of administration per prescription, dosage, number of points (medical expenses), flag for generics, flag for drugs to take as necessary, dosage form classification code
EF file: medical care activities	Medical care activities information relating to hospitalized patients and outpatients (data provided to MHLW)	Medical care activities date, medical care activities code, number of times performed, number of points (medical expenses)
EF file: treatment materials	Treatment material information relating to hospitalized patients and outpatients (data provided to MHLW)	Treatment date, material code, number of times performed, number of points (medical expenses)
H file	Nursing care requirement level information about hospitalized patients (data provided to MHLW)	Hospital admission date, discharge date, range in number of beds at hospitals, implementation date, nursing care requirement level of patients admitted to general wards, special intensive care wards, or high‐care units (wound treatment, pressure ulcer treatment, respiratory care, intravenous drip line, electrocardiographic monitoring, syringe pump use, blood transfusion, antitumor agent injection, oral antitumor agent administration, anesthetic injection, noninjection anesthetic administration, irradiation, immunosuppressive agents, vasopressor injection, anti‐arrhythmia agent injection, antithromboembolic agents, drainage, aseptic treatment room, admission after arrival as emergency, turning over in sleep, transfer movements, oral hygiene, taking meals, dressing and undressing, comprehension, dangerous actions, craniotomy, thoracotomy, laparotomy, bone surgery, thoracoscopic/laparoscopic surgery, surgery under general or spinal anesthesia, life‐saving percutaneous endovascular treatment, life‐saving percutaneous myocardial ablation, life‐saving invasive gastrointestinal treatment)
Clinical laboratory test values	Information about clinical laboratory test values (data from medical records)	Test date, hospitalized/outpatient classification, department classification code, test item code, result value, unit, abnormal value category, normal value range, JLAC10 code

## 3 DATA RESOURCE USE

The following analyses can be carried out using this database:

1. Detailed information about cancer and other important diseases is recorded on the DPC assessment forms. Therefore, it is possible to carry out an analysis after determining the results for medical care activities and dosage, depending upon disease state and severity, or, alternatively, the results for disease state and severity, depending upon medical care activities and dosage. In addition, using clinical laboratory test value data, it is possible to evaluate the treatment outcome/results based on dosage and medical care activities in such a way that the blood glucose control statuses of patients hospitalized for diabetes can be determined.
2. Unlike JMDC's another database of data from health insurance societies, this database includes elderly people, and it is therefore possible to calculate and analyze the medical expenses and numbers of days with hospital visits or admissions for diseases that frequently affect elderly people.
3. Hospitalized deaths can be used as endpoints when carrying out epidemiological studies, and even readmissions of the same patient to the same hospital can be ascertained and used in the analysis.

The most recent reference lists for this database of data from medical institutions are summarized on the publication lists page on the JMDC website [https://info.jmdc.co.jp/jrda/]. Please note that these lists correspond to two databases of JMDC, and currently, most are from the other database of data from health insurance societies.

## 4 STRENGTHS AND WEAKNESSES

This database is structural data in which DPC assessment forms are created based on the electronic medical record that doctors describe from a clinical point of view. It is useful for medical staffs to improve the management, the quality of medical care, and the clinical path, and also useful for researchers to derive new discoveries from case comparisons of diseases, because it is possible to immediately conduct case studies on this large database of all ages matching the actual situations of the clinical sites.

This database offers the following advantages:

1. Information about change in disease states, test results, etc., is included.
2. Numerous data about people aged over 65 are included.
3. Data from not only DPC‐eligible hospitals but also some DPC‐ineligible hospitals are included.

However, it has the following disadvantages:

1. Data cannot be confirmed to be from the same patient when the medical institutions are different.
2. Owing to the characteristics of medical institutions where data are collected, the numbers of long‐term care beds and psychiatric ward beds are small, and no data on clinics are available.
3. No information about the population is available.

**FIGURE 2:**
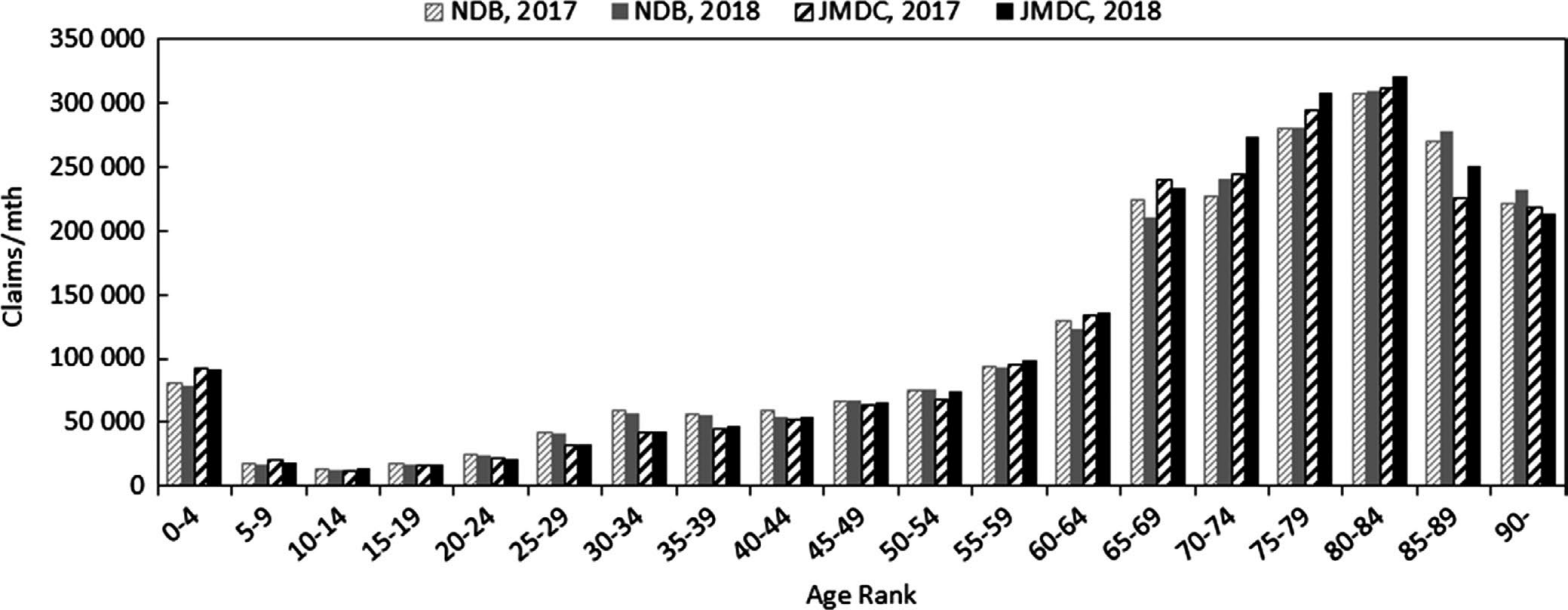
Comparison between scaled‐up data from medical institutions and publicly available data (numbers of hospitalized for each age rank)

**FIGURE 3:**
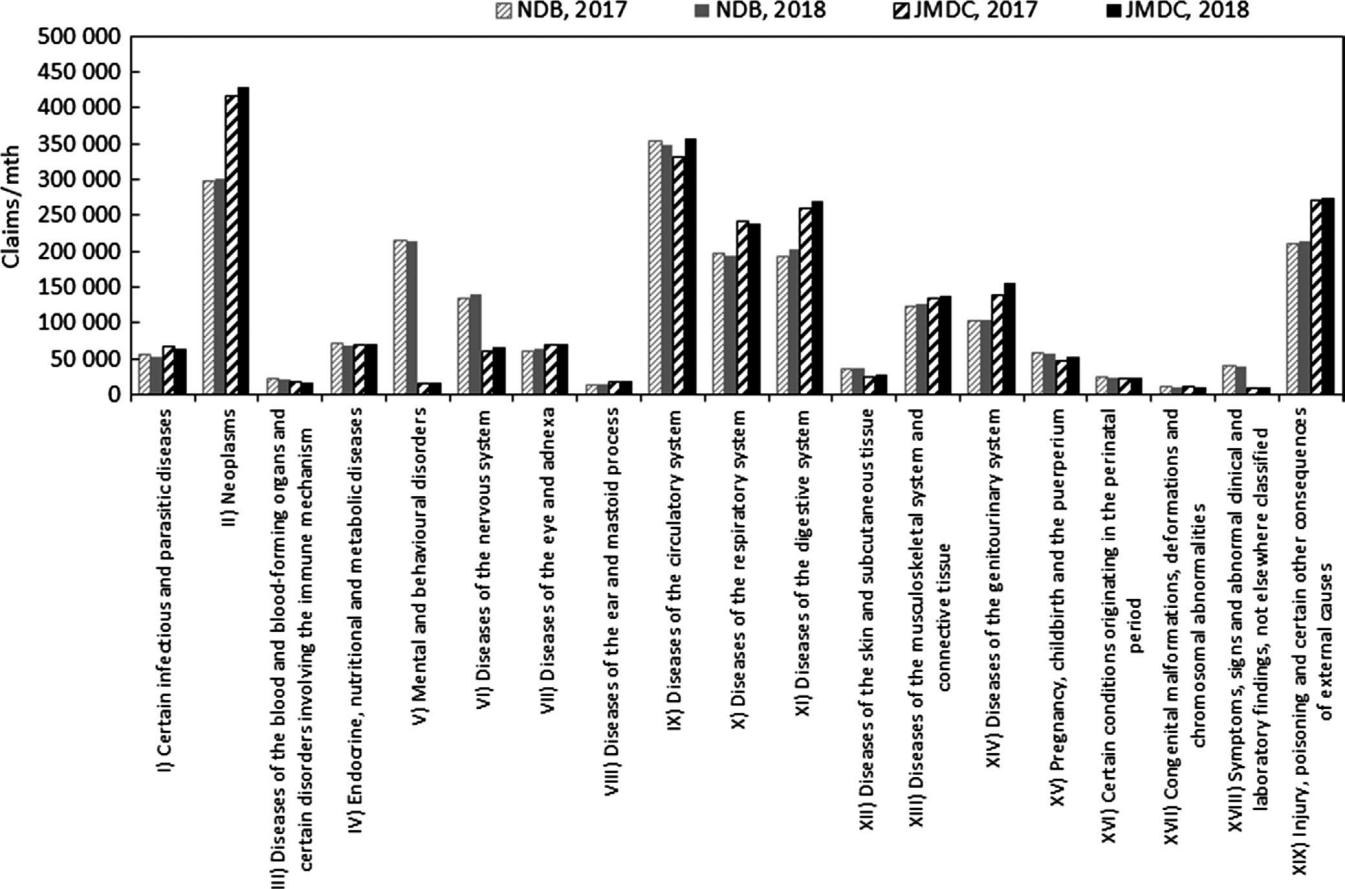
Comparison between scaled‐up data from medical institutions and publicly available data (numbers of hospitalized for each chapter of ICD‐10)

In order to quantify the above characteristics, comparisons were made with publicly available data. The publicly available data used were the numbers of claims in the June 2017 and June 2018 reviews in the MHLW's Statistics of Medical Care Activities in Public Health Insurance, which were aggregates of the National Database of Health Insurance Claims and Specific Health Checkups (NDB). In the case of hospitalization claims, the JMDC numbers of claims to be compared with them were obtained by scaling up the numbers of claims for treatment during May 2017 and May 2018 in this database of the data from medical institutions by the relative numbers of hospital beds in Japan and in medical institutions where data are collected at the end of October 2017 and October 2018. In this context, the publicly available data for the total number of hospital beds were taken to be the numbers of hospital beds, except for psychiatric ward beds, in the tables, titled "Numbers of institutions and beds, classified by type," for each month in the MHLW's Medical Institution Survey, as of end‐May each year. In other words, hospitalization at clinics is considered to be limited. The ratios used for scaling up, as database beds to total hospital beds of Japan, were about 3.3% at both the end of May 2017 and the end of May 2018. When two or more injuries/diseases were included in a single claim, the handling mode was similar to that with data from health insurance societies. In the case of hospitalization claims, the comparison results for each age rank are shown in Figure 2, and the comparison results for each chapter of ICD‐10 are shown in Figure 3.

**FIGURE 4:**
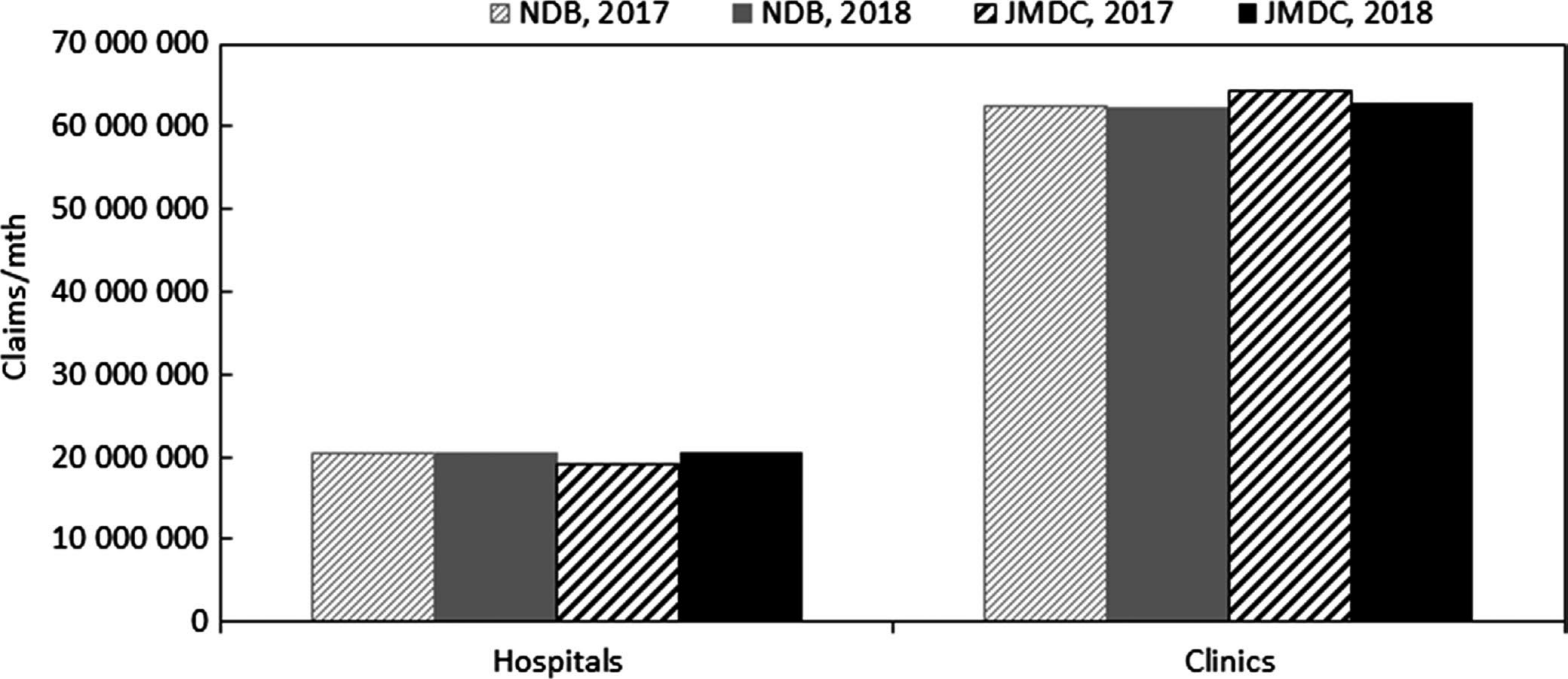
Comparison of the decomposition to hospitals and clinics between data from medical institutions scaled up to the total number of hospitals and publicly available data (numbers of outpatients)

Although the number of beds at clinics is considerably less than at hospitals, it is expected that the number of outpatients at clinics will be quite large. Therefore, scaling up by the relative number of beds is not considered to be appropriate according to the number of outpatients at clinics. Thus, the decomposition of hospitals and clinics in the publicly available total outpatient claims was compared between the publicly available numbers and the JMDC numbers calculated as follows. The JMDC number of outpatients at hospitals is the number of outpatients at hospitals in this database scaled up to the total number of hospitals using the same relative number of hospital beds, and the JMDC number of outpatients at clinics is the rest. Therefore, the total numbers of both hospitals and clinics constitute publicly available data. The comparison results of the decomposition are shown in Figure 4.

## 5 DATA RESOURCE ACCESS

JMDC makes its databases of data from medical institutions widely available, on a fee‐paying basis, for use of surveys, research, and commercial purposes. Inquiries about these databases can be made via JMDC's website [https://www.jmdc.co.jp/en/bigdata], and it can be accessed after completing the contract for use of specific data.

## 6 PROFILE IN A NUTSHELL

- JMDC has created a database from data on medical expenses, etc., collected from medical institutions.
- The earliest data that can be accessed are for April 2014. The number of persons whose data can be accessed for each month tends to increase from the initiation. Currently (the end of October 2019), the number of patients included in the database of data from medical institutions, including those withdrawn at some point, is approximately 9.4 million.
- In principle, all data collected from medical institutions are included in the databases. Nonstandardized data are standardized using a dictionary, and the database permits data to be followed in a time series on the basis of anonymized personal IDs.
- The database of data from medical institutions includes information about patients, claims, DPC assessment forms, and clinical laboratory test values.
- JMDC makes this database widely available, on a fee‐paying basis, for use for surveys, research, and commercial purposes. Inquiries about these databases can be made via JMDC's website [https://www.jmdc.co.jp/en/bigdata], and they can be accessed after completing the contract for use of specific data.

## 7 CONCLUSION

Using the database of data from medical institutions, it is possible to carry out an analysis that makes up for certain limitations of JMDC's another database of data from health insurance societies (eg, the disease status and test results cannot be ascertained, and there is insufficient access to data for elderly people). In addition, it is noteworthy that the database of data from medical institutions includes not only data from DPC‐eligible hospitals but also data from some DPC‐ineligible hospitals. Use of this database is on a fee‐paying basis. The characteristics mean that it is provided for a wide range of purposes.

### 7.1 GLOSSARY

#### 7.1.1 Medical institutions

This refers to all institutions that provide medical care. They are termed "hospitals" if they have 20 beds or more, and "clinics" if they have fewer than 20 beds.

#### 7.1.2 Claims

Certificates of payment of medical care fees, released by medical institutions, specifying the medical expenses for which health insurance providers (health insurance societies, etc.) are invoiced. They include information about injuries and diseases, medical care activities, drugs, etc.

#### 7.1.3 ICD‐10

The 10th version of the International Statistical Classification of Diseases and Related Health Problems, defined by the WHO.

#### 7.1.4 ATC

Anatomical Therapeutic Chemical classification systems, defined independently by the WHO and the European Pharmaceutical Market Research Association. Both the ATC systems are included in the JMDC's master data for classification.

#### 7.1.5 DPC assessment forms

Forms that the MHLW requests to be provided to medical institutions for evaluation of the level of effects, in the context of DPC, which is a medical expense calculation system that determines the daily hospitalization expenses for each diagnostic category. Form 1 contains detailed information about hospitalization, in a 'a medical records' format; the EF file contains itemized information about medical care fees; and the H file contains information about the nursing care requirement level.

#### 7.1.6 JLAC10

Version 10 of the Japanese Laboratory Code defined by the Japanese Society of Laboratory Medicine.

## CONFLICT OF INTEREST

Operation management of the database of data from medical institutions is conducted by JMDC. This article was co‐authored by academic researchers and JMDC Inc. (JMDC). First four authors are affiliated with JMDC. The other authors have no connection with JMDC.
